# A Barcoded ITS Primer-Based Nanopore Sequencing Protocol for Detection of *Alternaria* Species and Other Fungal Pathogens in Diverse Plant Hosts

**DOI:** 10.3390/jof11040249

**Published:** 2025-03-25

**Authors:** Vladimer Baramidze, Luca Sella, Tamar Japaridze, Nino Dzotsenidze, Daviti Lamazoshvili, Nino Abashidze, Maka Basilidze, Giorgi Tomashvili

**Affiliations:** 1 Department of Plant Protection, Agricultural University of Georgia, Kakha Bendukidze University Campus, Tbilisi 0159, Georgia; t.japaridze@agruni.edu.ge (T.J.); ndzocenidze@gmail.com (N.D.); dato.lamazoshvili@gmail.com (D.L.); 2 Microbiome Research Center, OxGEn Solutions, 14th km Natakhtari, Mtskheta 3308, Georgia; ninoabashidze34@gmail.com (N.A.); basilidzemaka@gmail.com (M.B.); 3 Department of Land, Environment, Agriculture and Forestry, University of Padua, 35020 Padova, Italy; luca.sella@unipd.it; 4 Department of Virology and Molecular Biology, National Center for Disease Control and Public Health (NCDC), Tbilisi 0198, Georgia; g.tomashvili@ncdc.ge

**Keywords:** *Alternaria* spp., barcoded ITS primers, nanopore sequencing, plant pathogen, mycobiome

## Abstract

*Alternaria* is a genus that contains several important plant pathogens affecting nearly 400 plant species worldwide, including economically important crops such as grapes, citrus, and ornamental plants. Rapid, scalable, and efficient methods of pathogen detection are crucial for managing plant diseases and ensuring agricultural productivity. Current amplicon sequencing protocols for *Alternaria* detection often require the enzymatic barcoding of amplicons, increasing hands-on time, cost, and contamination risk. We present a proof-of-concept study using custom barcoded primers, combining universal primers targeting ITS1 and ITS2 regions (600 bp) coupled with Oxford Nanopore Technologies (ONT) barcode sequences. Sequencing was performed on infected grapevine, mandarin orange, thuja, and maple tree samples. In total, we analyzed 38 samples using qPCR; 8 tested positive for *Alternaria*, which were sequenced using a newly developed protocol. As a result, we could identify *Alternaria* in every positive sample, and besides the pathogen of interest, we could identify the associated mycobiome. This protocol reduces hands-on time and cost, making a significant advancement over current sequencing methods. Future work will focus on optimizing our approach for high-throughput sequencing of up to 96 samples and determining the method’s applicability for large-scale mycobiome analysis.

## 1. Introduction

*Alternaria* is a significant plant pathogen, causing economic losses through reduced yield and quality [1]. The timely and cost-efficient identification of *Alternaria* species is critical, directly impacting plant management strategies [2]. Symptoms caused by *Alternaria* in plants typically include leaf spots, fruit rot, and stem lesions, which can lead to significant stress on the plants [3,4,5,6].

High-throughput sequencing (HTS) technologies have significantly transformed plant pathogen identification and mycobiome research fields [7]. Among available platforms, Oxford Nanopore Technologies (ONT) offers advantages such as affordable capital investment and portability for fieldwork [8]. ONT has shown great promise in plant pathogen diagnostics through several approaches, such as whole-genome sequencing (WGS) of the total nucleic acid content of the sample, including host plant DNA [9]. Adaptive sampling, an ONT technique aimed at selectively excluding the host genome during sequencing, is still in the developmental phase [10]. The depletion of host DNA can be achieved using several polynucleotide purification kits by selectively lysing the fungal and bacterial microorganisms [11]; however, such kits are only limited to human and animal host samples. Amplicon sequencing for fungal identification focuses on PCR amplification of internal transcribed spacers (ITS1 and ITS2) to avoid the limitation of WGS and enable the multiplexing of samples with unique molecular identifiers (UMIs) [12,13].

While standard UMI amplicon sequencing provides a convenient library preparation process, it still presents several limitations that can impact the efficiency of mycobiome research. Each sample necessitates amplicon DNA end-prep, enzymatic barcoding, and multiple steps of solid-phase reversible immobilization (SPRI) cleanup [14], which not only increases laboratory costs but also can lead to inefficiencies (loss of amplicons) and a heightened risk of amplicon fragmentation, which affects data quality. Also, every extra step increases the risk of aerosol contamination during library preparation.

Advancements in library preparation techniques that minimize the number of reaction steps can increase the sequencing data output. For instance, kits like the 16S Barcoding Kit 24 (SQL-16S024) from ONT allow the amplification of the 16S rRNA gene using barcoded primers. This method enables multiple samples to be pooled before the attachment of sequencing adapters. Additionally, several studies have been developed for amplicon-labeled DNA barcode strategies. For example, a study by Chen et al. (2023) [15] demonstrated the effectiveness of barcoded primers for bacterial 16S rDNA sequencing. In this study, barcodes were attached to the primers to allow for library preparation and sample pooling, which enabled rapid and cost-efficient pathogen identification in bacterial pneumonia cases. Similarly, Shaw et al. [16] utilized barcoded primers in ONT sequencing to detect polioviruses from stool and environmental samples. Another study by Leipart et al. [17] applied barcoded nanopore sequencing to analyze Vitellogenin protein variants in honey bees, revealing extensive diversity across samples. This study shows how barcoded primers enable successful amplification and multiplexing sequencing runs.

To date, the application of barcoded primer strategies, specifically for *Alternaria* species detection and associated mycobiome, still needs to be explored in ONT sequencing. In this paper, we introduce an *Alternaria* spp. detection method by sequencing the internal transcribed spacer (ITS) regions, specifically targeting ITS1, 5.8S, and ITS2, using the original barcodes of ONT attached to the primers. This is a proof-of-concept study that minimizes library preparation time and costs for fungal pathogen detection and mycobiome research.

## 2. Materials and Methods

### 2.1. Sample Collection

We collected a total of 38 symptomatic plant samples, exhibiting symptoms resembling *Alternaria* infection, from 4 distinct species, including 11 samples of *Vitis vinifera* (grapevine) and 11 samples of *Citrus reticulata* (mandarin), 9 samples of *Thuja* spp. (commonly known as thuja), and 7 samples of *Acer* spp. (maple). Samples were collected and provisionally diagnosed by the Mycology Department at the Agricultural University of Georgia.

The plant tissue samples exhibiting symptoms were harvested (berries or fruits for grapevine and mandarin, or leaves in the case of thuja and maple trees). Before DNA extraction, the plant samples’ surfaces were disinfected by immersing the samples in a 0.1% sodium hypochlorite solution for 30 s. Following this treatment, the samples were rinsed three times with sterile distilled water.

### 2.2. DNA Extraction and qPCR

The plant tissue samples exhibiting symptoms were sliced, including the berry skin and pulp for grapevine, peel for mandarin, leaf blade for maple trees, and scale leaves for thuja. Aseptically excised tissue fragments (200 to 400 mg) were taken on sterile scalpels and scissors

For DNA extraction, tissues were suspended in a pre-lysis buffer in homogenization tubes (OxGEn, Tbilisi, Georgia). Pre-lysed samples were homogenized with prefilled 0.7 mm ceramic beads, and DNA extraction was performed with the OxMag Pathogen DNA Purification Kit (OxGEn), according to the manufacturer’s protocol. The quality and quantity of the extracted DNAs were assessed using a NanoDrop ND-1000 spectrophotometer and Qubit 4 Fluorometer (Thermo Fisher Scientific, Waltham, MA, USA). PCR analysis was performed by the qPCR test kit (Youseq, Winchester, UK), following the specified guidelines provided by the manufacturer, to confirm the presence of *Alternaria* spp. The assay was carried out on the LineGene Mini S Fluorescent Quantitative Detection System (BIOER, Hangzhou, China). From the initial pool of 38 samples, 8 qPCR-positive and 4 negative samples for *Alternaria* spp. were selected for downstream sequencing applications.

### 2.3. Primer Design and PCR

Custom barcoded primers were developed by integrating native barcodes from ONT with universal primers targeting the internal transcribed spacer (ITS) gene region. Specifically, for the forward primers, barcodes numbered 1 through 12 from the EXP-NBD kit were attached to the 5′ terminus of the ITS1F primer (5′-CTTGGTCATTTAGAGGAAGTAA-3′). Similarly, for the reverse primers, the identical barcode sequences (not reverse complemented) were incorporated at the 5′ end of the ITS4 primer (5′-TCCTCCGCTTATTGATATGC-3′). A complete list of the resulting barcoded primer sequences is provided in Table S1. To evaluate potential issues related to self-complementarity and the formation of heterodimers among the primers, bioinformatic analysis was carried out using the Genius Prime software v2025.0 (Wellington, New Zealand). The effectiveness of the primer sets in amplifying the target was assessed by using an *Alternaria*-positive sample (Grape 01) with all 12 barcoded primer sets. As a control, we used the ITS1F and ITS4 primer set without barcodes (Figure S1).

The amplification of the target DNA was performed using primer pairs at 0.1 µM and an approximate total dsDNA template of 30 ng. The amplification process was performed using OneTaq^®^ Hot Start DNA Polymerase (New England Biolabs, Ipswich, NE, USA). The amplification reactions were carried out in a GeneExplorer Thermal Cycler (BIOER, Hangzhou, China) under the following thermal cycling conditions: an initial denaturation step at 94 °C for 30 s, followed by 30 cycles consisting of denaturation at 94 °C for 30 s, annealing at 53 °C for 45 s, extension at 68 °C for 1 min, and a final extension step at 68 °C for 10 min. Amplicons were visualized via electrophoresis on a 1% agarose gel.

### 2.4. Library Preparation

**Figure 1:**
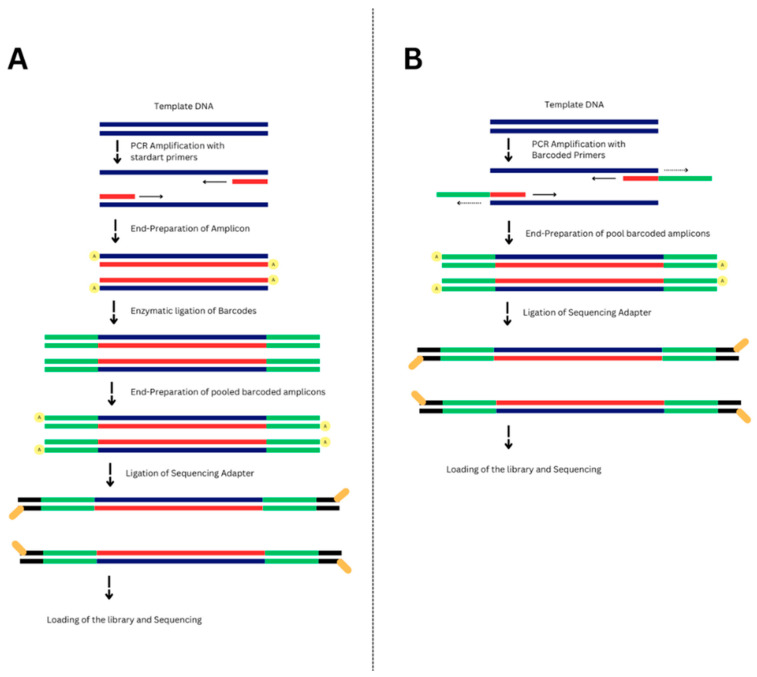
Schematic representation of (**A**) standard amplicon sequencing with ligation sequencing model for barcoding. (**B**) Newly developed protocol, without separate steps of barcode addition. The figure includes native DNA (blue lines), newly synthesized strand and oligonucleotides (red lines), nanopore barcodes (green lines), nanopore adapters (yellow lines), and the direction of PCR amplification (horizontal arrows).

In contrast to the standard ONT amplicon sequencing preparation workflow SQK-LSK114 (Oxford Nanopore, Oxford, UK), where barcodes are added via enzymatic ligation after PCR amplification (Figure 1A), our library preparation process involved a modified approach (Figure 1B). Firstly, barcoded amplicons were generated, followed by the direct attachment of adapters using the Blunt/TA ligase master mix (New England Biolabs, Ipswich, USA), which attaches sequencing adapters to the ends of the barcoded amplicons. All SPRI purification steps were performed using 1X OxMag XP beads (OxGEn, Tbilisi, Georgia).

The sequencing run was performed on the MinION Mk1B device (Oxford Nanopore, Oxford, UK). The sequencing run was made over 24 h, utilizing the MinKNOW software (v23.11.2).

### 2.5. Bioinformatics

The specialized bioinformatics pipeline was designed for the analysis of ITS sequences. For the demultiplexing of reads, the default configuration of Guppy software was changed in the barcode arrangement files. These files were made available through our GitHub repository and should be downloaded and placed in the appropriate directories within the Guppy installation following each software update or reinstallation. Within our GitHub repository, specifically in the folder labeled “Guppy_Customization”, we provided two modified files: ‘barcode_arrs_ITS.toml’ and ‘barcodes_masked.fasta’.

The classification of fungal reads was performed by the Guppy Aligner against a custom fungal ITS reference database (UNITE). To ensure the reliability of taxonomic assignments, we applied stringent filtering criteria, retaining only those reads that met a minimum coverage threshold of 70% and an identity threshold of 90%.

## 3. Results

Overall, 38 plant samples exhibiting putative *Alternaria* symptoms were visually assessed by the Mycology Department at the Agricultural University of Georgia. The samples were tested by a commercially available qPCR kit for *Alternaria* detection (Table S2). To assess the performance of barcoded primers, our study showed that amplification results were comparable across primer sets, showing similar band intensities and successful amplicon generation (Figure S1). In the future, we are planning to conduct comparative analytical sensitivity tests to identify the most effective primer sets.

We sequenced eight samples confirmed as *Alternaria*-positive (Ct values provided in Table S2). Additionally, for each plant sample, one qPCR-negative control was included in sequencing. The sequencing run resulted in 376,104 reads across all samples (total number of reads from 12 samples), with an average quality score of 11.2 and a read length of ~580 bp. The average number of reads per sample was 31,342, providing sufficient sequencing depth to accurately assess the fungal species composition within the complex mycobiota associated with field samples. The abundance of fungal species in thuja, maple, mandarin, and grape tissues is presented in Table 1.

**Figure 2:**
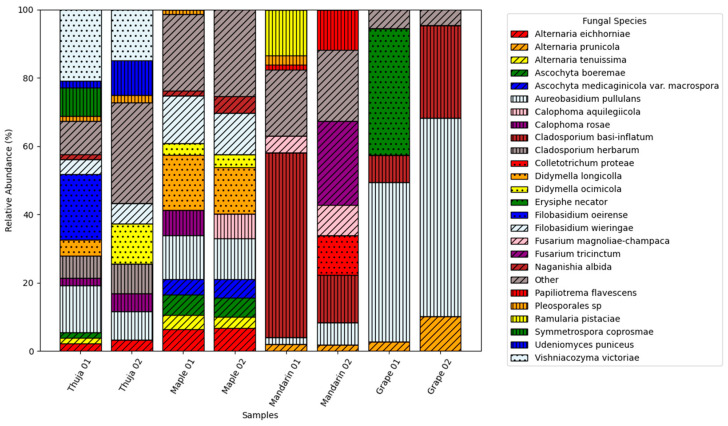
Relative abundance of the fungal species in different plant tissue samples above the 1% threshold.

The obtained results indicate that we could detect *Alternaria* in all the positive samples (Figure 2). To enhance the reproducibility of the reported information, we applied a 1% abundance threshold. *Alternaria prunicola* was detected in grapevine (Grape 01 and Grape 02) and mandarin (Mandarin 01 and Mandarin 02) samples, with abundances ranging from 1.29% to 9.71%. *Alternaria eichhorniae* was found in thuja (Thuja 01 and Thuja 02) and maple tree (Maple 01 and Maple 02) samples, with relative abundances between 1.66% and 6.13%. Similarly, *Alternaria tenuissima* was identified in thuja (Thuja 01) and maple tree (Maple 01 and Maple 02) samples, with abundances ranging from 1.21% to 3.35%. *Alternaria* spp. were not detected in the negative samples, thus showing the assay’s accuracy in *Alternaria* detection. The relative abundance of *Alternaria* spp. in the sequencing data was lower than that of other fungal species identified in the analyzed plant samples.

The complete list of detected fungal species in qPCR-positive and -negative samples is presented in Table S3. *Alternaria* species were not detected by way of sequencing in qPCR-negative samples. Analysis of fungal species distribution within the studied samples provides valuable insights into the composition of the fungal mycobiota. In thuja, *Vishniacozyma victoriae* was the dominant species (15.36%), followed by *Filobasidium oeirense* (14.11%). Maple samples showed predominance of *Didymella longicolla* (13.04%) and *Filobasidium wieringae* (11.41%). The mandarin samples exhibited different dominant species, with Mandarin 01 showing a high abundance of *Cladosporium basi-inflatum* (42.01%), while Mandarin 02 was dominated by *Fusarium tricinctum* (16.7%). The grape sample showed the highest abundance of *Aureobasidium pullulans* (45.58%), followed by *Erysiphe necator* (36.23%).

## 4. Discussion

We developed an optimized PCR barcoding protocol for the ONT detection of *Alternaria* spp. in different plant samples (citrus, grape, and ornamental plants). In total, we tested 38 samples because they visually appeared to be infected with *Alternaria*. However, qPCR analysis confirmed the presence of *Alternaria* spp. in only eight samples, and sequencing analysis further confirmed the absence of *Alternaria* species in the four selected qPCR-negative samples. *Alternaria* species consistently cause leaf spots, necrotic lesions, and blights. Our data suggest extensive symptom similarity with other pathogenic fungi identified in this study. For instance, *Fusarium* and *Colletotrichum* species cause necrotic lesions and blights similar to those caused by *Alternaria* [18,19]. *Ascochyta* and *Calophoma* species also show similar leaf spot patterns [20,21]. Members of the *Didymella* species also cause necrotic symptoms that are nearly impossible to differentiate from those resulting from *Alternaria* infections by visual inspection [22,23]. The extensive overlap of disease symptoms between various fungal genera [3,24,25] underscores the inadequacy of symptomatology and visual inspection alone for the identification/diagnosis of fungal pathogens.

The use of barcoded ITS primers allowed the efficient amplification and multiplexing of samples. We accurately identified *Alternaria* without false positives, along with other pathogenic and non-pathogenic fungi associated with each sample. Different species of *Alternaria* were detected, such as *A. prunicola*, *A. eichhorniae*, and *A. tenuissima*. *A. prunicola* has not been previously reported on citrus. The accuracy of the species-level identification could not be assessed since we used a general qPCR kit that did not differentiate *Alternaria* at the species level. Moreover, the *Alternaria* taxonomy is still controversial [26,27,28]. Therefore, we are not able to discuss how accurate the species-level identification of *Alternaria* is, and future studies are needed in this direction.

The identification of other fungal species, such as *Vishniacozyma victoriae* and *Filobasidium oeirense* in Thuja, as well as *Filobasidium wieringae* in maple, underscores the important role of endophytic fungi in plant health. These species are known to form symbiotic relationships with their hosts, potentially enhancing nutrient acquisition and stress resistance [29,30]. Interestingly, we also detected pathogens like *Fusarium tricinctum* in mandarin and *Erysiphe necator* in grape samples, which is the causal agent of grape powdery mildew, a significant disease that can cause yield losses and reduced fruit quality [31,32,33,34]. As for the grape samples, an interesting aspect is the abundance of *Aureobasidium pullulans*, a yeast species with dual ecological roles. While commonly found as an epiphyte, it also exhibits potential as a biocontrol agent against pathogens like *Botrytis cinerea* [35,36]. 

The methodology we developed employs custom-designed barcoded primers that specifically target the ITS1 and ITS2 regions, presenting a streamlined approach for the fungal pathogen diagnosis. By integrating barcodes directly into the PCR primers, we successfully eliminated the need for additional enzymatic barcoding, which reduces preparation time and minimizes the price and risk of contamination [37]. This enhancement significantly increases the protocol’s applicability for the high-throughput monitoring of *Alternaria* spp. and other plant-associated pathogens. The application of this protocol to different plant samples demonstrated its versatility across a range of host plants, achieving successful confirmation of *Alternaria* spp. within complex microbiomes. 

By utilizing barcoded primers during the PCR step, we were able to pool samples immediately post-amplification, applying end-prep reagents to a single pool instead of individual samples. This pooling strategy is not only cost-effective but also saves reagents, shortening the protocol. The final adapter ligation was performed with ONT’s Adapter Mix (AMX), which ensures compatibility with existing sequencing platforms for subsequent analyses.

Despite the promising outcomes of this study, certain challenges persist. Future analytical sensitivity tests are needed to identify which primer sets perform the best. Additionally, there is a need to investigate all 96 barcodes of the EXP-NBD kit. Moreover, we were not able to assess the accuracy of species-level identification of *Alternaria*, and future studies are needed in this direction.

## 5. Conclusions

In summary, the protocol we developed offers a rapid, scalable, and efficient method for the detection of the *Alternaria* genus in different plant hosts. The direct integration of barcoding into primer design reduces workflow complexity, making it well suited for both laboratory and field applications. By enabling high-throughput mycobiome analysis with minimal reagent consumption and preparation time, this method aligns with the growing demand for accessible and portable sequencing solutions for plant pathogen diagnostics. Beyond detecting the target pathogen, it also identifies other fungal species, highlighting its potential and wider applications in characterizing plant mycobiome and pathogen diagnostics.

## Figures and Tables

**Table 1:** Fungal abundance in different plant tissue samples (two samples, 01 and 02, for each plant species) above a 1% detection threshold.

Fungal Species	Thuja 01	Thuja 02	Maple 01	Maple 02	Mandarin 01	Mandarin 02	Grape 01	Grape 02
*Alternaria* spp. ^1^	2.87	2.79	8.56	9.11	1.59	1.29	2.68	9.71
Other Fungi ^2^	70.39	75.32	72.54	70.12	77.20	49.92	94.81	85.64

^1^ Combined abundance of the *Alternaria* species detected. ^2^ Other fungal species were detected in the plant samples analyzed.

## Data Availability

The original contributions presented in this study are included in the article and Supplementary Materials. Further inquiries can be directed to the corresponding author.

## Supplementary Materials

The following supporting information can be downloaded at the following link: https://www.mdpi.com/article/10.3390/jof11040249/s1, Table S1: Barcoded Primer Sequences Utilized in This Study containing ITS1f (forward) or ITS4 (reverse) sequences. The sequences reported in red correspond to ITS1f or ITS4; Table S2: This table provides the detailed qPCR diagnostic results for 38 plant samples tested for the presence of *Alternaria* spp.; Table S3: This table provides a comprehensive list of fungal species, expressed as a percentage of the total fungal population, detected across various plant samples, including Thuja, Maple, Mandarin, and Grapevine. Samples Thuja 01, Thuja 02, Maple 01, Maple 02, Mandarin 01, Mandarin 02, Grape 01, and Grape 02 represent *Alternaria* qPCR-positive samples, while Thuja 03, Maple 03, Mandarin 03, and Grape 03 represent *Alternaria* qPCR-negative samples. Species abundance is expressed as percentages, with a 1% threshold applied; Figure S1: Representative gel electrophoresis image of ITS amplification using non-barcoded and barcoded primers. Lane L1 contains the DNA ladder. Lane 1 shows amplification with the non-barcoded ITS primer. Lanes 2–13 represent amplification of the same sample using barcoded ITS primers (1–12). Lane NC is the negative control.
