# Supplementary material for: A Barcoded ITS Primer-Based Nanopore Sequencing Protocol for Detection of Alternaria Species and Other Fungal Pathogens in Diverse Plant Hosts
Source: J Fungi (Basel). 2025 Mar 25;11(4):249. doi: 10.3390/jof11040249 (PMC12027965; doi:10.3390/jof11040249)
Supplement: Supplementary file 1 [file jof-11-00249-s001.zip › jof-3372181-supplementary.pdf]

Supplementary Table S1. Barcoded Primer Sequences Utilized in This Study containing ITS1f (forward) or ITS4 (reverse) sequences. The sequences reported in red correspond to ITS1f or ITS4.

Primer	Sequences
BAF01F	CACAAAGACACCGACAACTTTCTTCTTGGTCATTTAGAGGAAGTAA
BAF01R	CACAAAGACACCGACAACTTTCTTTCCTCCGCTTATTGATATGC
BAF02F	ACAGACGACTACAAACGGAATCGACTTGGTCATTTAGAGGAAGTAA
BAF02R	ACAGACGACTACAAACGGAATCGATCCTCCGCTTATTGATATGC
BAF03F	CCTGGTAACTGGGACACAAGACTCCTTGGTCATTTAGAGGAAGTAA
BAF03R	CCTGGTAACTGGGACACAAGACTCTCCTCCGCTTATTGATATGC
BAF04F	TAGGGAAACACGATAGAATCCGAACTTGGTCATTTAGAGGAAGTAA
BAF04R	TAGGGAAACACGATAGAATCCGAATCCTCCGCTTATTGATATGC
BAF05F	AAGGTTACACAAACCCTGGACAAGCTTGGTCATTTAGAGGAAGTAA
BAF05R	AAGGTTACACAAACCCTGGACAAGTCCTCCGCTTATTGATATGC
BAF06F	GACTACTTTCTGCCTTTGCGAGAACTTGGTCATTTAGAGGAAGTAA
BAF06R	GACTACTTTCTGCCTTTGCGAGAATCCTCCGCTTATTGATATGC
BAF07F	AAGGATTCATTCCCACGGTAACACCTTGGTCATTTAGAGGAAGTAA
BAF07R	AAGGATTCATTCCCACGGTAACACTCCTCCGCTTATTGATATGC
BAF08F	ACGTAACTTGGTTTGTTCCCTGAACTTGGTCATTTAGAGGAAGTAA
BAF08R	ACGTAACTTGGTTTGTTCCCTGAATCCTCCGCTTATTGATATGC
BAF09F	AACCAAGACTCGCTGTGCCTAGTTCTTGGTCATTTAGAGGAAGTAA
BAF09R	AACCAAGACTCGCTGTGCCTAGTTTCCTCCGCTTATTGATATGC
BAF010F	GAGAGGACAAAGGTTTCAACGCTTCTTGGTCATTTAGAGGAAGTAA
BAF010R	GAGAGGACAAAGGTTTCAACGCTTTCCTCCGCTTATTGATATGC
BAF011F	TCCATTCCCTCCGATAGATGAAACCTTGGTCATTTAGAGGAAGTAA
BAF011R	TCCATTCCCTCCGATAGATGAAACTCCTCCGCTTATTGATATGC
BAF012F	TCCGATTCTGCTTCTTTCTACCTGCTTGGTCATTTAGAGGAAGTAA
BAF012R	TCCGATTCTGCTTCTTTCTACCTGTCCTCCGCTTATTGATATGC

Supplementary Table S2. This table provides the detailed qPCR diagnostic results for 38 plant samples tested for the presence of *Alternaria spp*.

Sample ID	Plant Species	qPCR Result	Ct Value
1	Grapevine (*Vitis vinifera*)	Positive	27
2	Grapevine (*Vitis vinifera*)	Negative	-
3	Grapevine (*Vitis vinifera*)	Negative	-
4	Grapevine (*Vitis vinifera*)	Negative	-
5	Grapevine (*Vitis vinifera*)	Negative	-
6	Grapevine (*Vitis vinifera*)	Negative	-
7	Grapevine (*Vitis vinifera*)	Positive	28
8	Grapevine (*Vitis vinifera*)	Negative	-
9	Grapevine (*Vitis vinifera*)	Negative	-
10	Grapevine (*Vitis vinifera*)	Negative	-
11	Grapevine (*Vitis vinifera*)	Negative	-
12	Mandarin Orange (*Citrus reticulata*)	Positive	30
13	Mandarin Orange (*Citrus reticulata*)	Negative	-
14	Mandarin Orange (*Citrus reticulata*)	Positive	26
15	Mandarin Orange (*Citrus reticulata*)	Negative	-
16	Mandarin Orange (*Citrus reticulata*)	Negative	-
17	Mandarin Orange (*Citrus reticulata*)	Negative	-
18	Mandarin Orange (*Citrus reticulata*)	Negative	-
19	Mandarin Orange (*Citrus reticulata*)	Negative	-
20	Mandarin Orange (*Citrus reticulata*)	Negative	-
21	Mandarin Orange (*Citrus reticulata*)	Negative	-
22	Mandarin Orange (*Citrus reticulata*)	Negative	-
23	Thuja (*Thuja spp.*)	Negative	-
24	Thuja (*Thuja spp.*)	Negative	-
25	Thuja (*Thuja spp.*)	Negative	-
26	Thuja (*Thuja spp.*)	Negative	-
27	Thuja (*Thuja spp.*)	Negative	-
28	Thuja (*Thuja spp.*)	Negative	-
29	Thuja (*Thuja spp.*)	Negative	-
30	Thuja (*Thuja spp.*)	Positive	29
31	Thuja (*Thuja spp.*)	Positive	30

32	Maple (*Acer spp.*)	Negative	-
33	Maple (*Acer spp.*)	Negative	-
34	Maple (*Acer spp.*)	Negative	-
35	Maple (*Acer spp.*)	Positive	29
36	Maple (*Acer spp.*)	Negative	-
37	Maple (*Acer spp.*)	Positive	35
38	Maple (*Acer spp.*)	Negative	-

Supplementary Table S3. This table provides a comprehensive list of fungal species, expressed as a percentage of the total fungal population, detected across various plant samples, including Thuja, Maple, Mandarin, and Grapevine. Samples Thuja 01, Thuja 02, Maple 01, Maple 02, Mandarin 01, Mandarin 02, Grape 01, and Grape 02 represent *Alternaria* qPCR-positive samples, while Thuja 03, Maple 03, Mandarin 03, and Grape 03 represent *Alternaria* qPCR-negative samples. Species abundance is expressed as percentages, with a 1% threshold applied.

**Fungal Species**	**Thuja 01**	**Thuja 02**	**Neg Thuja 03**	**Maple 01**	**Maple 02**	**Neg Maple 03**	**Mandarin 01**	**Mandarin 02**	**Neg Mandarin 03**	**Grape 01**	**Grape 02**	**Grape 03 Neg**
Acaromyces ingoldii							1.42	2.2	1.33			
Acrodontium neolitseae							1.15					
Alternaria eichhorniae	1.66	2.79		5.21	6.13							
Alternaria prunicola							1.59	1.29		2.68	9.71	

Alternaria tenuissima	1.21			3.35	2.98							
Ascochyta boeremae	1.11			4.85	5.32	3.46						
Ascochyta medicaginicola var. macrospora				3.69	4.97	3.25						
Ascomycota sp								1.01	5.28			
Aureobasidium pullulans	10.09	6.87		10.41	10.87		1.49	4.35	1.47	45.58	55.38	16.67
Botryosphaeria dothidea										5.4		2.59
Botrytis caroliniana							2.86		1.31		2.07	1.43
Buckleyzyma aurantiaca		2.13	4.75		1.87	2.63						
Buckleyzyma phyllomatis				1.23	1.55	4.75						
Bullera alba	1.18		2.13									

Calophoma aquilegiicola					6.75	4.38						
Calophoma clematidina				1.83		4.38						
Calophoma rosae	1.55	4.31		5.99								
Cladosporium basi-inflatum							42.01	9.43		7.6	25.83	3.36
Cladosporium herbarum	4.88	7.21	10.41									
Colletotrichum nupharicola								1.37	18.59			
Colletotrichum proteae								7.98				
Colletotrichum psidii								1.55	1.31			
Colletotrichum roseum					2.11	2.78						
Colletotrichum								1.17				

xanthorrhoeae												
Didymella chromolaenae					3.16							
Didymella longicolla	3.33			13.04	12.43	9.14						
Didymella ocimicola		9.78		2.72	3.48	2.87						
Ectophoma multirostrata		3.22		2.68								
Epicoccum brasiliense					3.94							
Epicoccum pimprinum				2.32								
Erysiphe necator										36.23		13.40
Erythrobasidium hasegawae							1.29					
Exobasidiomycetes sp							3.51		1.42			

Filobasidium floriforme	1.99	2.46	7.06									
Filobasidium oeirense	14.11		4.12									
Filobasidium wieringae	3.19	4.99	3.11	11.41	11.22	4.16						
Fungi sp	2.73							2.86	1.31			
Fusarium magnoliae-champaca							3.89	5.93				
Fusarium nurragi								1.23				
Fusarium tricinctum								16.68	1.53			
Holtermannia saccardoi		5.43	3.11									
Naganishia albida	1			1.15	4.52	4.86						
Naganishia globosa		5.92	3.20									

Neodidymelliopsis cannabis				5.62		4.38						
Neodidymelliopsis sp				1.51	2.39	2.78						
Nothophoma ngayawang					2.57							
Papiliotrema flavescens							1.1	8.01	1.50			
Phaeococcomyces sp					5.81							
Pleosporales sp	1.13	1.84		1.07		4.38	2.14		1.47			
Ramularia pistaciae							10.49		18.59			
Setophaeosphaeria badalingensis							1.62		1.44			
Sporobolomyces patagonicus											2.37	1.53

Strelitziana albiziae								1.56				
Symmetrospora clarorosea				3.02								
Symmetrospora coprosmae	6.09											
Symmetrospora suhii								1.27				
Symmetrospora symmetrica		3.87	2.84									
Taphrinaceae sp	1.24											
Trichomerium dioscoreae		1.52	4.45									
Udeniomyces puniceus	1.41	8.34	4.85									
Vishniacozyma tephrensis							3.14		1.36			

Vishniacozyma victoriae	15.36	12.45	10.41								

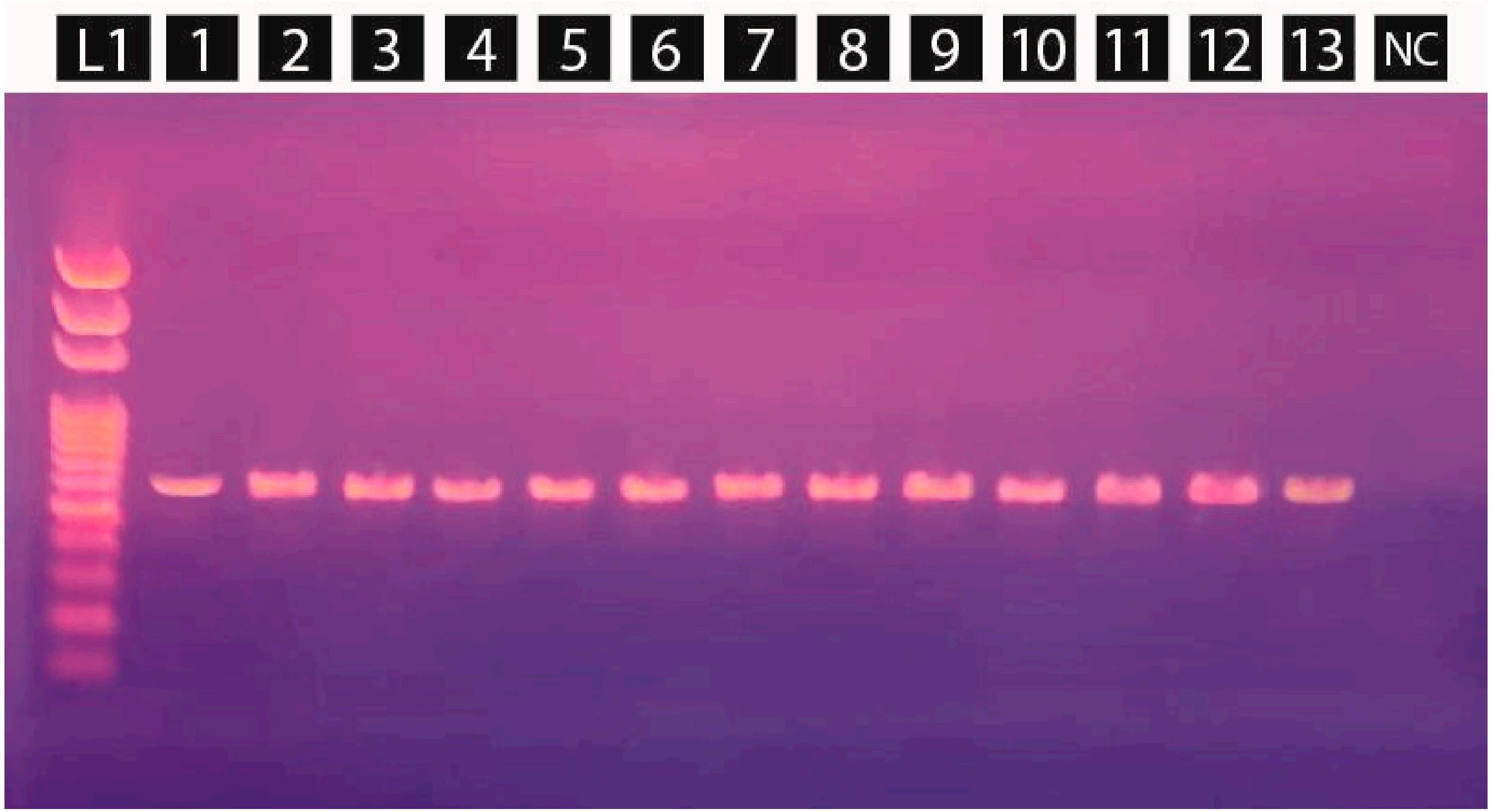

Supplementary Figure S1. Representative gel electrophoresis image of ITS amplification using non-barcoded and barcoded primers. Lane L1 contains the DNA ladder. Lane 1 shows amplification with the non-barcoded ITS primer. Lanes 2–13 represent amplification of the same sample using barcoded ITS primers (1–12). Lane NC is the negative control.
